# Identification of Reference Genes in Chicken Intraepithelial Lymphocyte Natural Killer Cells Infected with Very-virulent Infectious Bursal Disease Virus

**DOI:** 10.1038/s41598-020-65474-3

**Published:** 2020-05-22

**Authors:** Sook Yee Boo, Sheau Wei Tan, Noorjahan Banu Alitheen, Chai Ling Ho, Abdul Rahman Omar, Swee Keong Yeap

**Affiliations:** 1 Laboratory of Vaccines and Immunotherapeutic, Institute of Bioscience, Universiti Putra Malaysia, 43400 Serdang, Selangor Malaysia; 2 Faculty of Biotechnology and Biomolecular Sciences, Universiti Putra Malaysia, 43400 Serdang, Selangor Malaysia; 3 China-ASEAN College of Marine Sciences, Xiamen University Malaysia, Bandar Sunsuria, 43900 Sepang, Selangor Malaysia

**Keywords:** Reverse transcription polymerase chain reaction, Transcriptomics

## Abstract

Due to the limitations in the range of antibodies recognising avian viruses, quantitative real-time PCR (RT-qPCR) is still the most widely used method to evaluate the expression of immunologically related genes in avian viruses. The objective of this study was to identify suitable reference genes for mRNA expression analysis in chicken intraepithelial lymphocyte natural killer (IEL-NK) cells after infection with very-virulent infectious bursal disease virus (vvIBDV). Fifteen potential reference genes were selected based on the references available. The coefficient of variation percentage (CV%) and average count of these 15 genes were determined by NanoString technology for control and infected samples. The M and V values for shortlisted reference genes (*ACTB*, *GAPDH*, *HMBS*, *HPRT1*, *SDHA*, *TUBB1* and *YWHAZ*) were calculated using geNorm and NormFinder. *GAPDH*, *YWHAZ* and *HMBS* were the most stably expressed genes. The expression levels of three innate immune response related target genes, *CASP8*, *IL22* and *TLR3*, agreed in the NanoString and RNA sequencing (RNA-Seq) results using one or two reference genes for normalisation (not *HMBS*). In conclusion, *GAPDH* and *YWHAZ* could be used as reference genes for the normalisation of chicken IEL-NK cell gene responses to infection with vvIBDV.

## Introduction

Infectious bursal disease affects the poultry industry and has a significant impact on the economics of some countries^1^. It is important to understand the early immune response of chicken infected by vvIBDV, so that prevention steps can be taken to avoid the spread of the virus within the farm^2^. One of the approaches to understanding the innate immune response of the chicken towards vvIBDV is performing gene expression studies using the cost-effective RT-qPCR method. However, the reliability and accuracy of RT-qPCR results mainly depends on the reference genes used to normalise the expression level of the target gene^3^. Reference genes selected for expression studies are supposed to be constitutively expressed in specific tissues or cells. However, it has been found that expression level of some commonly used reference genes, such as beta actin (*ACTB*) or glyceraldehyde-3-phosphate dehydrogenase (*GAPDH*), can be altered in different types of biological samples and experimental conditions^4^. For example, there is of concern to molecular virologists because most viruses regulate key biological and molecular processes, which may change the expression of common reference genes. Different types of viruses manipulate different cellular transcription-related pathways, and the extent to which pathways are affected will be dependent on the infected cell type and virus strain. Thus, it is not possible to identify a reference gene that should be used for expression studies on all virus-infected cell types^5^.

One of the ways to overcome this problem is to assess the stability of candidate reference genes for specific biological samples or experimental conditions^5^. Some studies have been carried out to determine the best reference genes for chicken embryo cells infected by IBDV^6^, H5N1 avian influenza virus (AIV)^7^, Newcastle disease virus (NDV)^5^ and Avian leukosis virus subgroup J^8^. However, there is not a single reference gene that is stable across different types of virus infection in the same cell type, i.e. chicken embryo cells in this case. Therefore, it is important to carry out an experiment to find out the best reference genes for chicken intraepithelial lymphocyte natural killer (IEL-NK) cells infected with vvIBDV strain UPM0081. The IEL-NK was classified as CD3^-^28.4^+^ cells because it can be isolated out by using CD3 and 28.4 markers according to the previous study^9^. There are many new technologies that can be used for expression studies, such as NanoString and RNA-Seq technology, which allow for the detection of the expression levels of a large number of genes in a single run. NanoString technology is similar with the microarray which relies on the hybridization of target on the probes whereby the probes target the gene of interest. As compared to NanoString, NGS has advantage on novel discovery as the whole transcriptome can be sequenced and hypothesis free. NanoString and RNA-Seq have been used for reference gene selection in previous studies^10–14^. In this study, NanoString RNA-Seq and RT-qPCR were utilised to get a better view of the expression profile of reference genes and to identify the best reference genes.

## Results

Chicken (4 weeks old) infected with 10^3^ EID_50_ of vvIBDV strain UPM0081 survived until day 3 of the experiment. Clinical signs including diarrhea, feather ruffling and loss of appetite were observed in the infected chicken starting 3 days post-infection (dpi). The IEL-NK cells were isolated from duodenum samples collected from uninfected chicken and chicken infected by vvIBDV at 1 and 3 dpi. The total number of IEL-NK cells is 19.48 million for control samples, 14.29 and 13.95 million cells for chicken infected for 1 day and 3 days. After the enrichment process using the CD3 and 28.4 markers, the percentage of CD3^-^28.4^+^ IEL-NK cells at 1 dpi and 3 dpi are higher than uninfected control group, which are 37.82% and 42.38% (Table 1).

**Table 1:** Total IEL cells, percentage of IEL-28.4^+^ cells and total no of IEL-28.4^+^ cells.

	Control	Day 1	Day 3
Total no. of IEL cells/chicken (million)	19.48 ± 2.34	14.29 ± 2.84*	13.95 ± 3.38*
% of IEL-28.4^+^ cells	33.61 ± 2.23	37.82 ± 3.21*	42.38 ± 2.12*
Total no of IEL-28.4^+^ cells/chicken (million)	6.43	5.29*	5.86*

Results from three biological replicates (n = 3). One biological replicate of each sample consisted of IEL cells or IEL-28.4^+^ cells harvested from 8 chickens.

Percentage of IEL-28.4^+^ cell population was quantified by flow cytometry while total number of IEL cells was counted post-harvesting of IEL cells. * indicates significant between different samples using one-way ANOVA (p < 0.05). The value was the means ± SEM of three experiments.

Reporter Code Count (RCC) files were loaded into the nSolver^TM^ Analysis Software version 4.0 for data quality checking, spike-in-control normalisation and reference gene normalisation. All the NanoString QC tests passed, which indicated there is no issue with the starting material and processing steps. Then, the raw data proceeded to gene expression analysis. The coefficient of variation percentage (CV%) and average counts were calculated for all the genes in the panel. The CV% and average count from NanoString for 15 reference genes are shown in Table 2. Among 15 candidate reference genes, *YWHAZ* had the lowest CV% of 27.68% and *B2M* had the highest CV% of 65.91%. For the average count number, *ACTB* had the highest expression level (96,218 counts), followed by *UB* (70,600 counts). Seven genes, i.e. *ACTB*, *GAPDH*, *HMBS*, *HPRT1*, *SDHA*, *TUBB1* and *YWHAZ* fulfilled the requirements were selected for reference gene selection using the RT-qPCR approach.

**Table 2:** Average count and CV% from NanoString for 15 candidate reference genes.

Gene	Average count	CV%
*ACTB*	96,218	31.04
*ALB*	19	36.63
*B2M*	15,986	65.91
*G6PDH*	688	42.00
*GAPDH*	16,943	38.22
*HMBS*	1,444	35.80
*HPRT1*	715	34.67
*IFNA3*	28	52.21
*RPL4*	15,044	44.96
*RPL30*	13,605	48.41
*SDHA*	4,325	31.65
*TBP*	350	41.40
*TUBB1*	1,998	38.27
*UB*	70,600	40.55
*YWHAZ*	4,085	27.68

Results from three biological replicates (n = 3). One biological replicate of each sample consisted of IEL-28.4^+^ cells harvested from 8 chickens.

The efficiency of the seven primer pairs used for RT-qPCR were determined by generating a standard curve. The primer sequences, amplicon size and efficiency% are listed in Table 3. The stability of seven reference genes was determined with the analysis software geNorm and NormFinder. The geNorm and NormFinder results are summarised in Table 4. According to the geNorm results, all the references genes were considered stable as the M values were less than 1.5. The reference gene with lowest M value was *GAPDH*, followed by *YWHAZ* and *HMBS*. Meanwhile, there were two reference genes considered as stable genes based on the NormFinder results, which are *HMBS* and *YWHAZ*.

**Table 3:** Primer sequences for target and reference genes used for RT-qPCR.

	Symbol	Forward primer (5’ to 3’)	Reverse primer (5′ to 3′)	Amplicon size (bp)	RT-qPCR Efficiency (%)
Reference genes	*ACTB*	GCTACAGCTTCACCACCACA	TCTCCTGCTCGAAATCCAGT	90	106.8
	*GAPDH*	GCAGATGCAGGTGCTGAGTA	GACACCCATCACAAACATGG	144	102.2
	*HMBS*	GACTGACAGCGTGGTTGAGA	CCAGCTCTTTGGTGAAGAGG	143	102.7
	*HPRT1*	AAGTGGCCAGTTTGTTGGTC	GTAGTCGAGGGCGTATCCAA	110	95.8
	*SDHA*	TTCCCGTTTTGCCTACGGTG	CTGCCTCGCCACAAGCATAT	126	101.8
	*TUBB1*	CCGCATCAGCGTCTACTACA	GTCTGAAGATCTGCCCGAAG	125	102.9
	*YWHAZ*	TTCTTGATCCCCAATGCTTC	TTGTCATCTCCAGCAGCAAC	104	97.1
Target genes	*CASP8*	GGTGAGCAGCAAGATTGACA	CTGCCTCTGCTCCCATTTAG	110	102.2
	*IL22*	CAGGAATCGCACCTACACCT	CGGTTGTTCTCCCTGATGTT	108	107.2
	*TLR3*	CCCTGATGGAGTGTTTGCTT	CCAGGGTTTTGAAAGGATCA	95	116.9

**Table 4:** Stability value of seven candidate reference genes based on result from geNorm and NormFinder.

Reference genes	geNorm (M value)	NormFinder (V value)
*YWHAZ*	0.62	0.14
*ACTB*	0.96	0.57
*SDHA*	0.66	0.33
*HPRT1*	0.78	0.46
*HMBS*	0.65	0.08
*GAPDH*	0.58	0.17
*TUBB1*	1.1	0.7

Results from three biological replicates (n = 3). One biological replicate of each sample consisted of IEL-28.4^+^ cells harvested from 8 chickens.

**Figure 1:**
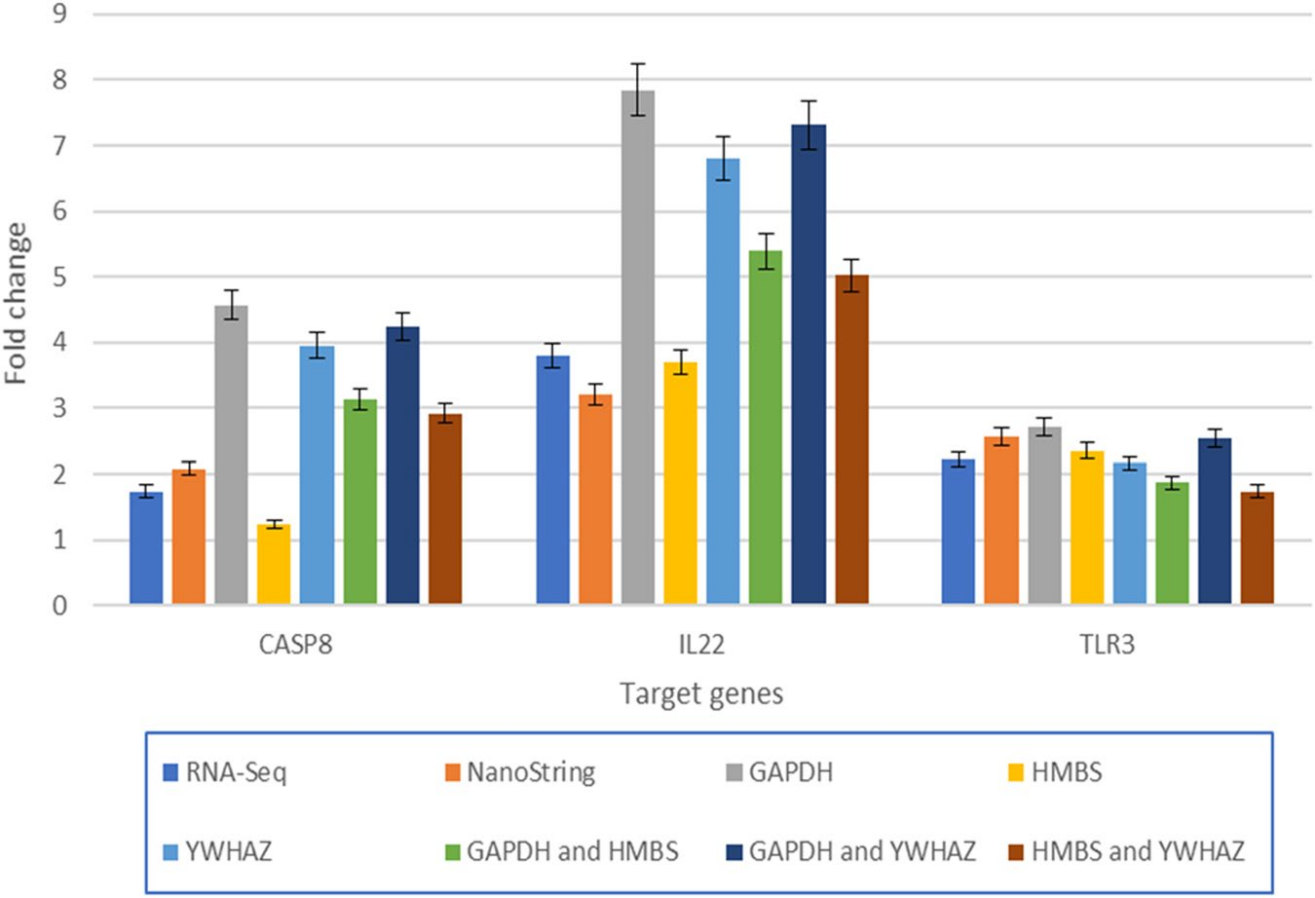
Comparison of RNA-Seq and NanoString results for target genes with RT-qPCR results using different combinations of reference genes. All these differential expression results were generated by comparing chicken IEL-NK cells infected with vvIBDV at 3 days post-infection with cells from uninfected chickens. Results from three biological replicates (n = 3). One biological replicate of each sample consisted of IEL-28.4^+^ cells harvested from 8 chickens.

Three reference genes with the highest stability based on geNorm and NormFinder results, i.e. *GAPDH*, *HMBS* and *YWHAZ*, were selected to be used as reference genes for an expression study on three target genes related to the innate immune response (*CASP8*, *IL22* and *TLR3*). These three genes were selected from a list of differentially expressed genes detected in RNA-Seq and NanoString studies (unpublished data). According to the RNA-Seq and NanoString results (Table 5), these three target genes were differentially up-regulated at 3 days post infection (dpi) (fold change > 1.5 and P-value < 0.05) in chicken CD3^−^28.4^+^IEL-NK cells infected with vvIBDV. The fold changes in the target genes normalised with different combinations of reference genes are summarised in Fig. 1. The fold change value in Figure 1 was summarized in supplementary file 1, 2 and 3.

**Table 5:** Fold change and P-value for target genes (*CASP8*, *IL22*, *TLR3*) based on the results from RNA-Seq and NanoString.

Genes	Description	RNA-Seq		NanoString	
		Fold change	P-value	Fold Change	P-value
*CASP8*	Caspase 8	1.74	0.008	2.08	0.000
*IL22*	interleukin 22	3.79	0.009	3.21	0.002
*TLR3*	toll like receptor 3	2.22	0.020	2.57	0.001

Results from three biological replicates (n = 3). One biological replicate of each sample consisted of IEL-28.4^+^ cells harvested from 8 chickens.

^*^The control samples were compared with 3 dpi samples.

In general, the expression levels of the three target genes based on RT-qPCR are in agreement with the expression levels from NanoString and RNA-Seq, which were up-regulated with a fold change greater than 1.5. The fold changes of *CASP8*, *IL22* and *TLR3* were higher than 1.5 when normalised with *GAPDH* and *YWHAZ*, but the fold change in *CASP8* was 1.2 when *HMBS* was used for the normalisation of expression data. When two reference genes were used for RT-qPCR data normalisation, all the target genes showed a fold change of more than 1.5, which indicated that the expression data were more reliable when more reference genes were used for RT-qPCR data normalisation. The reference gene combination of *GAPDH* and *YWHAZ* showed the lowest mean M value of 0.32 and CV% of 0.11 (Table 6), so this combination of reference genes will be used for RT-qPCR result normalisation in future studies.

**Table 6:** Mean M value and CV for different combination of reference genes used for target gene normalization.

	Mean M value	Coefficient Variance (CV)
*GAPDH* and *HMBS*	0.55	0.19
*GAPDH* and *YWHAZ*	0.32	0.11
*HMBS* and *YWHAZ*	0.48	0.17

Results from three biological replicates (n = 3). One biological replicate of each sample consisted of IEL-28.4^+^ cells harvested from 8 chickens.

## Discussion

Many studies have been carried out to determine the best reference genes, which are constitutively expressed in different cell types or under different environmental conditions. The most common methods used to identify the best reference genes is through RT-qPCR, followed by stability analysis using software such as geNorm and NormFinder. As newly available technologies such as NanoString and next generation sequencing (NGS) become more affordable, more studies will be conducted to screen for the stability of a larger number of reference genes within a short period of time. In this study, NanoString technology was utilised to check the stability of 15 candidate reference genes. According to a previous study^10^, the NanoString method is more accurate than RT-qPCR due to fewer sample handling steps, no sample amplification steps and the direct measurement of target molecules, thus avoiding any bias. In this study, the CV% were positively correlated with the M and V values generated from geNorm and NormFinder software, which indicates that the NanoString CV% can be used as the initial stability screening for a large number of reference genes.

Viruses are obligate parasites that replicate inside cells and use various strategies to induce cell transformation, apoptosis and cell death by manipulating gene expression on different scales. Thus, the expression of common RT-qPCR reference genes in virus infected chicken cells is unstable, but this depends on the virus strain and host cell type^8^. Some studies have been carried out to identify the best reference genes to be used in chicken cell types infected by different types of viruses such as H5N1 AIV^15^, Newcastle disease virus^16^, avian leukosis virus subgroup J^8^ and IBDV^6^. Based on the results of existing studies, there are no reference genes with stable gene expression in chicken embryo fibroblasts infected with different viruses; thus, it is recommended to determine the best reference genes when dealing with different types or strains of virus.

In the present study, *GAPDH* and *YWHAZ* were found to be the most stable reference genes for the RT-qPCR assay in chicken IEL-NK cells infected with vvIBDV. *YWHAZ* has been being reported as one of the best reference genes in chicken embryo fibroblast cells infected by H5N1 AIV^7^. In that study, there were 11 reference genes (*ALB, B2M, GAPDH, HRPT1, RPL30, RPL4, SDHA, TBP, TUBB, YWHAZ* and *ACTB*) examined, and the geNorm results showed that *RPL4* and *YWHAZ* were the most stable reference genes to be used for chicken cells infected with H5N1 AIV.

Other than the unstable expression caused by virus infection, the normalisation of gene expression data in lymphocytes such as IEL-NK cells is very challenging because the activation of lymphocytes completely changes the metabolism of these cells and affects processes such as cell proliferation, differentiation, and the secretion of cytokines; infection can also lead to the expression of new surface antigens on lymphocytes. Several studies on lymphocytes in human and mouse showed that commonly used reference genes have variable gene expression. Bas and coworkers^17^ found that the expression of two commonly used reference genes, *ACTB* and *GAPDH*, increased upon human T cell activation. Other than human T lymphocytes, a study carried out on macrophages differentiated from primary mouse bone marrow monocytes also showed that two common reference genes, *18S rRNA* and *GAPDH*, showed variable gene expression, whereas *HMBS* and *B2M* were the most stable genes^18^. Another study carried out by the Dheda team found that, in peripheral blood mononuclear cell (PBMC) cultures stimulated with tuberculosis antigen, the most stable genes were *HuPO* and *HPRT* whereas *GAPDH*, *B2M*, *ACTB* and *EIF-1-α* (elongation factor 1-α)^4^ were less stable genes.

In our studies, geNorm and NormFinder classified *ACTB* as less stable reference gene, but *GAPDH* was one of the most stable reference genes. Although many studies have shown that *GAPDH* has variable gene expression in lymphocytes, another study conducted by Kaszubowska and colleagues^19^ showed that *GAPDH* is one of the best reference genes for human NK-92 cell lines stimulated with IL-2 or TNF for 2, 24 or 72 hours. Thus, *GAPDH* and *YWHAZ* could be used as reference genes for the normalisation of chicken IEL-NK cell gene response following infection with vvIBDV, whereas the commonly used *ACTB* is unsuitable to be used as a reference gene in IEL-NK cells infected with vvIBDV.

It is important to validate the shortlisted reference genes by analysing the expression profile of two or three target genes with known expression levels. In this study, *CASP8*, *IL22* and *TLR3* were chosen to be used as target genes to assess the performance of the shortlisted reference genes, because these three genes play important roles in the innate immune response against virus infection. CASP8 induces inflammation and apoptosis after infection by a virus^20^. One study has shown that the expression level of *CASP8* was increased at 24, 48 and 72 hours post-infection by IBDV, using *GAPDH* as the reference gene^21^. The RNA-Seq and NanoString results (unpublished) showed that the expression of *CASP8* was significantly up-regulated at 3 dpi, a similar finding to previous studies. Additionally, *CASP8* was up-regulated when *GAPDH* and *YWHAZ* were used to normalise the RT-qPCR results, which showed that these two genes are appropriate as reference genes.

IL-22 has been found to regulate mucosal epithelial function by maintaining barrier integrity and protecting from bacterial and virus infection in the gut and lung^22–24^. One study has shown that *IL-22* is up-regulated in human hepatoma cell lines after infection by hepatitis C virus^25^. In this study, *IL-22* was up-regulated at 3 dpi based on NanoString, RNA-Seq as well as RT-qPCR results normalised with three shortlisted reference genes. The expression of *TLR3* was detected in immune cells such as dendritic cells, macrophages and fibroblasts for dsRNA virus recognition. It induces the production of interferon and initiates signalling pathways, such as the NFκB and the MAPK cascades, which result in the expression of pro-inflammatory mediators after virus infection^26^. *TLR3* is upregulated in most studies related to IBDV^27,28^. In the present study, *TLR3* was up-regulated according to the RNA-Seq, NanoString and RT-qPCR results at 3 dpi; the upregulation of this gene induces the production of cytokines and interferons for protection against vvIBDV.

According to the expression profile of these three target genes, it showed that the innate immune system of the infected chicken was activated and start to fight against the virus by triggering several pathways such as apoptosis, toll like receptor and cytokine-cytokine receptor pathway. According to the study conducted by our previous study^9^, the expression of *B-Lec*, one of the activator receptor on the NK cells was up-regulated at 2 and 3 dpi after infected by vvIBDV which indicated the NK cell was activated. This concordance with the finding in this study which three genes related to innate immune response were activated at 3 dpi.

On the other hand, it is suggested to have more than one reference gene being used for the normalization step for RT-qPCR result. In some of our previous studies^9,29,30^, one reference gene was being used as the reference gene for RT-qPCR data analysis which is *GAPDH*. This is because *GAPDH* is known to be a stable reference in some of the chicken related research^31–33^. However, the Fig. 1 in this study showed that *CASP8*, *IL22* and *TLR3* have the highest expression level when the *GAPDH* was used as reference genes which indicated that it is possible the expression level of target gene might be overestimated when *GAPDH* was being used for normalization purpose.

This is the first study to identify the best reference genes for expression studies in chicken IEL-NK cells infected with vvIBDV. The combination of *GAPDH* and *YWHAZ* was the best for normalisation as it provided an accurate expression level for the target genes and showed high stability based on the CV%, M and V values.

## Materials and Methods

### Ethical considerations

Based on the reference number of UPM/IACUC/AUP-R051/2014, all animal experiments were approved by local animal care authority and pathogenicity study by the Institutional Animal Care and Use Committee (IACUC), Faculty of Veterinary Medicine, UPM following ethical guidelines for the care and use of lab animals by the committee.

### Chickens and viruses

Nine-day-old SPF embryonated chicken eggs were purchased from Veterinary Research Institute, Ipoh, Perak and incubated under sterile conditions at Laboratory of Vaccines and Immunotherapeutics, Institute of Bioscience (IBS), Universiti Putra Malaysia (UPM). Once hatched, the chickens were transferred to the biosafety level-2 (BSL-2) animal house facility where they were fed with pelleted feed and supplied with water *ad libitum*. The vvIBDV strain UPM0081 was kindly provided by Prof Dr. Abdul Rahman Omar from IBS, UPM, Malaysia.

Seventy-two SPF chickens at 4 weeks of age were randomly divided into three groups (i.e. 24 chickens in each group); control group (without viral infection/healthy group); chicken infected with vvIBDV for 1 day; and chicken infected with vvIBDV for 3 days. Chickens from the infection groups were challenged with the vvIBDV strain UPM0081 stock at a dosage of 10^3^ EID_50_ in a volume of 0.1 ml through eye-nose drops. The chickens of the control group were treated with 1× phosphate-buffered saline (PBS), pH 7.4. All of the chickens were housed under 12-hours dark-light cycle and sterilised tap water and standard pellet-diet were provided throughout the study. At 1 and 3 dpi, 24 chickens of each group were randomly selected and killed under anaesthesia using carbon dioxide for duodenum collection. The duodenal loops were harvested and submerged in sterile-cold Roswell Park Memorial Institute (RPMI) 1640 medium (R6504, Sigma, USA) before IEL isolation was carried out.

### CD3^−^28.4^+^IEL-NK cell isolation

The isolation of IEL-NK cells from duodenum was carried out as described by Jahromi^9^. About 2 × 10^8^ isolated IEL cells were resuspended in 100 ul PBS-BSA-EDTA buffer and 10 μl of CD3 Phycoerythrin (PE) mAb (8200-09, Southern Biotech, USA) was added. The CD3^−^ cells that passed through the MACS BS column were collected and then labelled with 28.4 mAb (a kind gift by Prof. Thomas Göbel, Germany). The purity and quantity of the CD3^−^28.4^+^ IEL-NK cells were measured using a flow cytometer (BD FACSCalibur, San Jose, CA). All CD3^−^28.4^+^ IEL-NK cells obtained from eight chickens were pooled together as one biological replicate, giving a total of three biological replicates per group.

### RNA extraction and mRNA isolation

Total RNA was extracted from IEL-NK cells using the Trizol method following the manufacturer’s instructions and checked for RNA integrity number to inspect RNA integrity by an Agilent 2100 Bioanalyzer (Agilent technologies, Santa Clara, CA, US). The mRNA was isolated from total RNA using the NEBNext Poly(A) mRNA Magnetic Isolation Module (E7490S, NEB, USA) following the manufacturer’s instructions. The mRNA samples were used for NanoString, RNA-Seq and lastly for RT-qPCR assay.

### NanoString® nCounter assay

Fifteen potential reference genes were selected based on existing studies^6–8,15,16,34–36^. The list of reference genes is listed in Table 7. NanoString nCounter Elements probes for 15 reference genes were designed by the NanoString Bioinformatics team and synthesised by Integrated DNA Technologies (IDT) in Singapore. Triplicate total RNA samples extracted from uninfected chickens, chickens infected by vvIBDV for 1 and 3 days were being used for the NanoString assay. Briefly, a total of 200 ng of mRNA was used for NanoString assay. Samples were incubated for 16 to 21 hours at 67 °C as stated in the manufacturer’s protocol to ensure hybridisation with capture probes and reporter. After the hybridisation step, the samples were processed inside the Prep Station (NanoString Technologies, Seattle, WA) and counted in the Digital Analyser (NanoString Technologies, Seattle, WA). Reporter Code Counts (RCC) files were generated by the Digital Analyser system and loaded to the nSolver^TM^ Analysis Software version 4.0 (NanoString Technologies, Seattle, WA) for data quality checking, spike-in-control normalisation and reference gene normalisation. Furthermore, the percentage of CV for each reference gene was calculated.

**Table 7:** List of reference genes being tested in NanoString.

Symbol	Gene name	Accession number	Targeted position
*ACTB*	Beta-actin	NM_205518.1	1172-1271
*ALB*	Albumin	NM_205261.2	1049-1148
*B2M*	Beta-2-microglobulin	NM_001001750.1	116-215
*G6PDH*	glucose-6-phosphate dehydrogenase	AI981686.1	153-252
*GAPDH*	Glyceraldehyde-3-phosphate dehydrogenase	NM_204305.1	186-285
*HMBS*	Hydroxymethylbilane synthase	XM_417846.2	776-875
*HPRT1*	Hypoxanthine phosphorribosyltransferase 1	NM_204848.1	486-585
*IFNA3*	Interferon alpha 3	NM_205427.1	207-306
*RPL4*	Ribosomal protein L4	NM_001007479.1	971-1070
*RPL30*	Ribosomal protein L30	NM_001007967.1	147-246
*SDHA*	Succinate dehydrogenase complex, subunit A	NM_001277398.1	900-999
*TBP*	TATA box binding protein	NM_205103.1	1015-1114
*TUBB1*	Beta-tubulin class I	NM_205315.1	1859-1958
*UB*	ubiquitin	M11100.1	444-543
*YWHAZ*	Tyrosine 3-monooxygenase/tryptophan 5-monooxygenase activation protein, zeta polypeptide	NM_001031343.1	1175-1274

### RNA-Seq

The library preparation was conducted using ScriptSeq v2 library prep kit (SSV21106, Epicentre, USA) by following the manufacturer’s instruction with modifications. At least 50 ng of mRNA was used for library preparation. The quality and quantity of the final library were checked using the Qubit, qPCR and the bioanalyser. The final libraries were loaded into the cBot system (Illumina, San Diego, USA) for cluster generation and followed by sequencing (2 ×101 cycles) in the HiSeq. 2500 (Illumina, San Diego, USA) according to standard protocols. The data for each sample was not less than 25 million reads and the percentage of Q30 is more than 90%. After the sequencing was completed, the obtained bcl files were transformed into fastq files using bcl2fastq software. FastQC was used to check the quality of the reads and adapter dimer contamination. The raw reads were loaded into the CLC Genomics Workbench and cleaned to remove the low-quality reads, including adapter sequences shorter than 50 nt with Q < 30 at the 3′ end. The clean reads were mapped to the chicken genome (galGal4, Ensembl release 85). The differentially expressed (DE) genes were identified with fold changes ≥2 and FDR corrected P-value <0.05. The raw sequencing data and processed data files have been deposited in the Gene Expression Omnibus (GEO) at the NCBI under accession number of GSE123920.

### cDNA synthesis

mRNA was reverse transcribed into cDNA using NEXscript^TM^ cDNA synthesis kit (NexQ-R50, NEX^TM^ Diagnostics, Korea). Briefly, 1 μl of 50 μM Oligo dT and 100 ng of mRNA were added together and the volume was topped up to 10 μl. The mixture was kept for 5 mins at 65 °C and immediately chilled on ice. After that, the template RNA and primer mix were added with 4 μl of 5x reaction buffer, 1 μl of RTase enzyme (200 U/μl) (NexQ-R50, NEX^TM^ Diagnostics, Korea) and 1 μl of 2.5 mM dNTP (NexQ-R50, NEX^TM^ Diagnostics, Korea). The total volume was adjusted to 20 μl using nuclease-free water. The mixture was placed in a thermocycler at 50 °C for 60 mins for cDNA synthesis and followed by 95 °C for 5 mins in order to inactivate the reverse transcriptase enzyme. The final cDNA product was used for the RT-qPCR run.

### RT-qPCR and data analysis

Based on the NanoString results, reference genes with CV% less than 40% and average counts more than 500 were selected to proceed for the RT-qPCR. A previous study^32^ found that genes with a low expression level will not be chosen as candidate reference genes because of low amplification efficiency and accuracy. Therefore, we excluded genes with low average counts for further stability analysis. RT-qPCR was carried out on a BioRad CFX96 Real Time PCR System (BioRad Laboratories, Hercules, CA) using a QuantiNova SYBR Green PCR kit (208056, Qiagen, Hilden, Germany). RT-qPCR was carried out in a total reaction volume of 20 μl containing a final concentration of 1x QuantiNova SYBR Green PCR Master Mix, 50 nM of each forward and reverse primer and 1 µl of cDNA. Thermal cycling conditions were designed as follows: initial denaturation at 95 °C for 2 mins, followed by 40 cycles of 95 °C for 5 seconds and 60 °C for 10 seconds. A non-reverse transcriptase control (NRT) control in triplicate were included in each RT-qPCR run.

The stability of each of the reference genes was analysed using NormFinder^37^ and geNorm^38^ analysis tools, which were also able to rank reference genes based on their expression stability value (M value) and pairwise variation value (V value), respectively. The cut-off M value of 1.5 and V value of 0.15 was applied to geNorm and NormFinder separately^37,38^. A M value generated by geNorm less than 1.5 is considered as stable, whereas a reference gene with a V value lesser than 0.15 with NormFinder is considered stable. The M value and CV% of the combinations of two reference genes were calculated from BioRad CFX manager v3.0^39^. The expression level of three target genes, i.e. *CASP8*, *IL22* and *TLR3*, were examined using the three reference genes with the lowest M and V values for normalisation purposes.

## Supplementary information

Supplementary Dataset 1.

Supplementary Dataset 2.

Supplementary Dataset 3.

## Data Availability

The authors declare that all the data in this manuscript are available.
